# *Ex Vivo* Treatment with a Novel Synthetic Aminoglycoside NB54 in Primary Fibroblasts from Rett Syndrome Patients Suppresses *MECP2* Nonsense Mutations

**DOI:** 10.1371/journal.pone.0020733

**Published:** 2011-06-13

**Authors:** Manuela Vecsler, Bruria Ben Zeev, Igor Nudelman, Yair Anikster, Amos J. Simon, Ninette Amariglio, Gideon Rechavi, Timor Baasov, Eva Gak

**Affiliations:** 1 Sagol Neuroscience Center, Sheba Medical Center, Tel Hashomer, Israel; 2 Sackler Faculty of Medicine, Tel Aviv University, Tel Aviv, Israel; 3 Edmond and Lilly Safra Pediatric Hospital, Sheba Medical Center, Tel Hashomer, Israel; 4 The Edith and Joseph Fisher Enzyme Inhibitors Laboratory, Schulich Faculty of Chemistry, Technion – Israel Institute of Technology, Haifa, Israel; 5 Cancer Research Center, Sheba Medical Center, Tel Hashomer, Israel; University of Kent, United Kingdom

## Abstract

**Background:**

Nonsense mutations in the X-linked methyl CpG-binding protein 2 (*MECP2*) comprise a significant proportion of causative *MECP2* mutations in Rett syndrome (RTT). Naturally occurring aminoglycosides, such as gentamicin, have been shown to enable partial suppression of nonsense mutations related to several human genetic disorders, however, their clinical applicability has been compromised by parallel findings of severe toxic effects. Recently developed synthetic NB aminoglycosides have demonstrated significantly improved effects compared to gentamicin evident in substantially higher suppression and reduced acute toxicity *in vitro*.

**Results:**

We performed comparative study of suppression effects of the novel NB54 and gentamicin on three *MECP2* nonsense mutations (R294X, R270X and R168X) common in RTT, using *ex vivo* treatment of primary fibroblasts from RTT patients harboring these mutations and testing for the C-terminal containing full-length MeCP2. We observed that NB54 induces dose-dependent suppression of *MECP2* nonsense mutations more efficiently than gentamicin, which was evident at concentrations as low as 50 µg/ml. NB54 read-through activity was mutation specific, with maximal full-length MeCP2 recovery in R168X (38%), R270X (27%) and R294X (18%). In addition, the recovered MeCP2 was translocated to the cell nucleus and moreover led to parallel increase in one of the most important MeCP2 downstream effectors, the brain derived neurotrophic factor (BDNF).

**Conclusion:**

Our findings suggest that NB54 may induce restoration of the potentially functional MeCP2 in primary RTT fibroblasts and encourage further studies of NB54 and other rationally designed aminoglycoside derivatives as potential therapeutic agents for nonsense *MECP2* mutations in RTT.

## Introduction

Rett syndrome (RTT, MIM 312750) is a postnatal neurodevelopmental disorder predominantly occurring in girls with a worldwide incidence of 1/10,000–15,000 female births [1]. Classical RTT patients suffer from profound cognitive and motor disabilities usually apparent after the first year of life. In addition, the majority of RTT patients also develop seizure disorder, and various autonomic dysfunctions including breathing abnormalities, sleep disorder and orthopedic complications. Loss of purposeful hand use and emergence of stereotypic hand movements are the hallmark of RTT. The major causative factor of RTT is deficiency of the methyl CpG binding protein *MECP2* at Xq28 [2], in which over 200 mutations have been identified so far in classical and atypical RTT patients [3]. The majority of RTT causative mutations involve C>T transitions at the CpG hot-spots leading to missense, nonsense and frame-shift mutations [4], mostly originating *de novo* in the paternal germline [5]. Phenotypic heterogeneity in RTT has been related, for the most part, to *MECP2* mutation type and localization, as well as X chromosome inactivation (XCI) pattern [6]. However, not only MeCP2 deficiency but also its overdose is equally detrimental for the CNS, as *MECP2* gene duplications have been found in patients reminiscent of RTT [7], [8].

The *MECP2* gene encodes two isoform proteins, MeCP2_e1 and _e2 products of an alternative initiation at exon 1 and splicing of exon 2 [9], [10], both of which are nuclear and co-localize with the methylated heterochromatin [11]. Previous studies suggested that MeCP2 is a classical transcriptional repressor binding to methylated promoters and recruiting the HDAC machinery to induce chromatin condensation [12], [13]. In neurons, MeCP2 has been implicated in modulation of specific neuronal target genes in activity dependent manner, specifically the brain derived neurotrophic factor (BDNF) [14], [15]. However, more recent studies proved that MeCP2 role in neurons is more flexible and complex, as MeCP2 has been implicated in both repression and activation of a large number of genes [16], in modulation of RNA splicing [17], and most recently has been suggested to affect global chromatin structure impacting on the entire neuronal genome [18]. An important realization learned from RTT mouse models was that MeCP2 dysfunction in mature neurons accounts for RTT symptoms [19], [20] and that postnatal restoration of MeCP2 deficiency in the CNS, even after RTT onset, can lead to the reversal of neurological symptoms [21], [22]. These findings have lead to the notion that RTT rescue may be achieved by pharmacological treatment that may induce MeCP2 up-regulation in MeCP2 deficient neurons, nonetheless considering the importance of correct MeCP2 dosage [23].

Significant proportion (up to 60%) of the classical RTT is caused by *MECP2* nonsense mutations [24], leading to premature translational termination and truncated protein products. Aminoglycoside antibiotics, such as gentamicin, can induce suppression of nonsense codons in mammalian cells by enabling partial read-through and expression of functional proteins [25], [26], [27]. Partial suppression effect of gentamicin was demonstrated *in vitro* and *in vivo* for specific nonsense mutations related to human genetic disorders [28], [29], [30]. Previous study using recombinant MeCP2 constructs harboring the most common RTT nonsense mutations, R168X, R255X, R270X and R294X, showed that gentamicin can recover MeCP2 read-through efficiency up to 10–22% depending on the nucleotide context of a nonsense mutation [31]. In addition, the recovered MeCP2 protein was traced to the cell nucleus suggesting that gentamicin does not interfere with its nuclear localization. However, clinical applicability of gentamicin has been compromised by parallel findings of significant toxicity associated with its long-term administration and with reduced suppression efficiency at subtoxic doses [30], in addition to its limited permeability through the blood-brain-barrier [32]. Synthetic aminoglycosides, NB aminoglycosides, developed by systematic structure-activity-toxicity design optimized for maximal suppression effect and minimal toxicity [33], could be potential candidates for nonsense suppression therapy in RTT. Experience with the NB30 derivative suggested that it can induce significant read-through of the p.R31X nonsense mutation related to Usher's syndrome with better biocompatibility and significantly reduced toxicity compared to gentamicin and paromomycin [34]. The newer NB54 compound demonstrated even lesser acute toxicity and significantly higher suppression potency [35].

We presently report on experiments with NB54 and gentamicin treating primary fibroblasts cultures derived from female RTT patients harboring common *MECP2* nonsense mutations, R294X, R270X or R168X. We considered that non-transformed fibroblasts may provide a better experimental system for comparative studies *ex vivo* of NB54 and gentamicin effects, avoiding limitations of the transformed or transfected cell lines or unavailability of complex tissues such as brain. We presently show that NB54 has better read-through efficiency than gentamicin for MeCP2 harboring RTT causative nonsense mutations, as determined by recovery of the C-terminal containing MeCP2 protein. In addition, we show that NB54 treatment restores potentially functional MeCP2, which is evident from its appropriate nuclear localization and increased levels of BDNF.

## Results

### Efficiency of NB54 treatment compared to gentamicin

**Figure 1:**
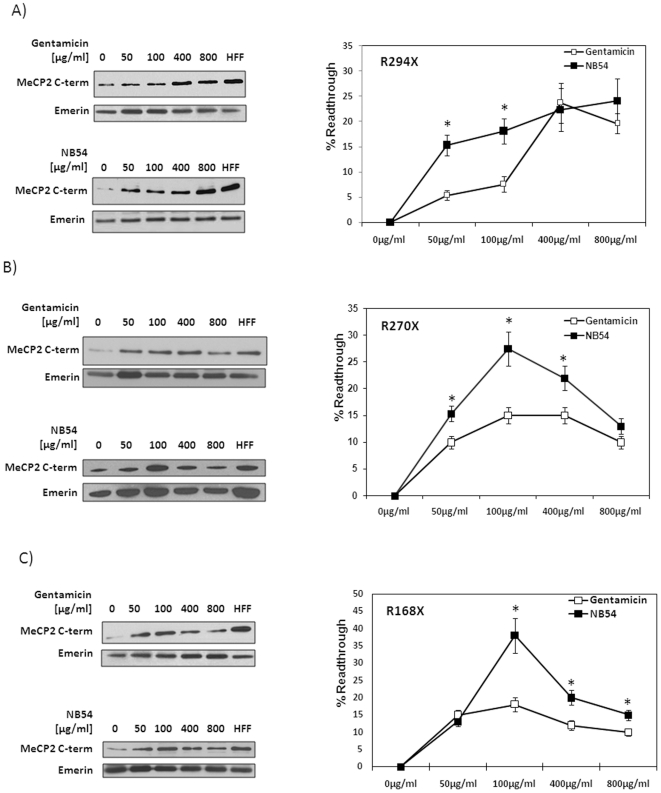
Aminoglycosides produce full-length MeCP2 in primary RTT fibroblasts. Western blot analyses of nuclear protein extracts from gentamicin- and NB54-treated (50–800 µg/ml for 5days) and untreated RTT fibroblasts. 20 µg of nuclear protein extract were loaded into each lane; full-length MeCP2 was detected using anti-MeCP2 C-terminal antibody. Graphs indicated mean ± SEM of read-through efficiencies that were determined using full length MeCP2 densitometric readings at each point normalized to emerin for protein load correction, subtracting the no-drug point in each experiment for correction of MeCP2 background expression and XCI variability and dividing by human foreskin fibroblasts (HFF) reference for fully active wild type MeCP2 (n = 3 independent experiments). Statistical significance by Student's t test is indicated (*). (A) R294X (t = 5.8, p<0.05), (B) R270X (t = 2.6, p<0.05), (C) R168X (t = 2.7, p<0.05).

We performed experiments in primary RTT fibroblasts harboring R168X, R270X or R294X mutations, treating them in parallel with NB54 or gentamicin at concentrations ranging from 50 to 800 µg/ml for duration of 5 days. Expression levels of the full-length MeCP2 (approximately 75 kD) was evaluated using MeCP2 C-terminal antibody and emerin as nuclear protein reference; MeCP2 read-through efficiency was evaluated relative to the human foreskin fibroblasts (HFF) expressing the normal MeCP2. Figure 1 shows NB54 and gentamicin effects on R294X (Fig. 1A), R270X (Fig. 1B) R168X (Fig. 1C) RTT fibroblasts, relating to the full-length MeCP2 expression levels and MeCP2 read-through efficiency. Read-through efficiency was calculated considering full length MeCP2 readings at each point normalized to emerin for protein load correction, subtracting the no-drug point in each experiment for correction of MeCP2 background expression and XCI variability, and dividing by HFF reference for fully active wild type MeCP2. In all RTT fibroblasts, NB54 treatment was more effective than gentamicin, enabling to achieve higher read-though efficiency and higher MeCP2 expression levels. Maximal MeCP2 read-though of 38% was detected in R168X treated with 100 µg/ml NB54, compared to 18% - with gentamicin at the same concentration (Fig. 1C). NB54 effect in R270X and R168X fibroblasts was similar, with maximal MeCP2 read-through of 27% and 38% at 100 µg/ml, respectively, and decreased efficiency in higher drug concentrations (Fig. 1B and C). Gentamicin had similar effects at the same concentration in R270X and R168X fibroblasts, but significantly lesser MeCP2 read-through of 15% and 18%, respectively (Fig. 1B and C). In R294X fibroblasts, NB54 had dose-dependent effect in increasing read-through efficiency between 15% and 24% at increasing drug concentrations from 50 to 800 µg/ml (Fig. 1A). Gentamicin showed different kinetics for the same mutation with maximal read-through of 24% at 400 µg/ml and decreased efficiency at a higher concentration (Fig. 1A). Basal levels of the full-length MeCP2, in the range of 25–50% (relative to HFF), were detected in all RTT fibroblasts (Fig. 1A–C), which result from XCI variability in primary cultures reflecting the expression of the active MeCP2 allele. In all RTT fibroblasts, NB54 effect was apparent at concentrations as low as 50 µg/ml in increasing up to 15% MeCP2 read-through, in addition, NB54 was tolerated at higher concentrations compared to gentamicin. However, both aminoglycosides demonstrated a degree of cell toxicity (LC50) even during short-term treatment (48 h) with gentamicin and NB54 in HEK293 and HFF cells (Table 1).

**Table 1:** Cytotoxicity of gentamicin and NB54 in HEK293 and HFF cells.

Aminoglycoside^a^	Cell toxicity LC50 (mg/ml)^b^	
	HEK293	HFF
**gentamicin**	1.73±0.35	2.1±0.20
**NB54**	3.56±0.39	4.54±0.24

Aminoglycoside-induced cell toxicity was measured in human embryonic kidney cells (HEK293) and in human foreskin fibroblasts (HFF) treated for 48 h.

a Aminoglycosides are sulfate salts (MW_gentamicin_ = 653.21 and MW_NB54_ = 652.81) and concentrations refer to the free amine form.

b Cell toxicity was calculated as ratio between the number of living cells in the presence of aminoglycoside *versus* non-treated cultures. The half-maximal lethal concentration (LC50) values were obtained from fitting concentration–response curves to the data obtained from at least three independent experiments, using GraFit 5 software.

### Efficiency of NB54 in various RTT nonsense mutations

**Figure 2:**
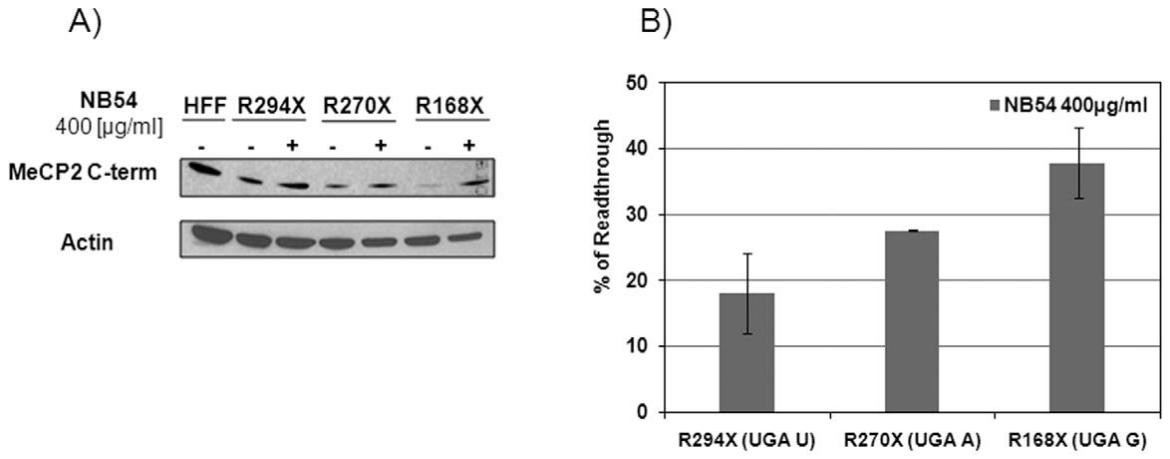
Aminoglycoside NB54 enhances read-through in primary RTT fibroblasts. (A) Western blot analysis of NB54-treated (400 µg/ml for 5days) and untreated RTT fibroblasts with R168X (CGA G>UGA G), R270X (CGA A>UGA A) and R294X (CGA T>UGA U) mutations, using MeCP2 C-terminal antibody. 30 µg of nuclear protein extract were loaded into each lane. Read-through efficiency was determined using full length MeCP2 densitometric readings normalized to actin for protein load correction, subtracting the no-drug point in each experiment for correction of MeCP2 background expression and XCI variability, and dividing by human foreskin fibroblasts (HFF) reference for fully active wild type MeCP2 (n = 3 independent experiments); (B) Effect of NB54 treatment was quantified by densitometric developed Western blot analysis (n = 3 independent experiments).

**Figure 3:**
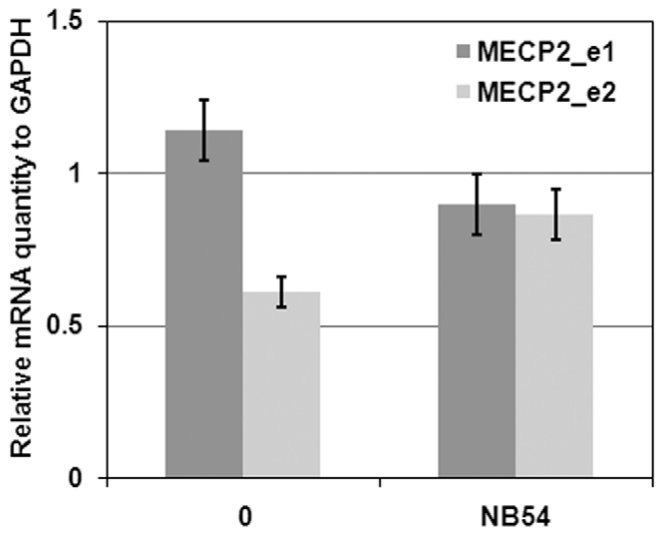
MECP2 mRNA expression levels following treatment with NB54. Total mRNA was purified from NB54-treated (100 µg/ml for 5days) and untreated R294X fibroblasts. Expression levels of both *MECP2*_e1 and _e2 isoforms were determined by real-time qPCR using *GAPDH* levels as an internal reference. Experiments were performed in triplicates.

Mutations specific effects of NB54 were further reproduced treating R294X, R270X and R168X fibroblasts with 400 µg/ml NB54 for 5 days (Fig. 2A and B) that was previously found optimal in R294X (Fig. 1A). This experiment independently demonstrated that NB54 was more effective in R168X (UGA G), with increased 38% read-through compared to 18% and 27% in R294X (UGA U) and R270X (UGA A), respectively. The difference between this and previous findings (Fig. 1) of NB54 effect at the concentration of 400 µg/ml may be an outcome of XCI variability. Studying expression *MECP2* RNA levels in R294X fibroblasts treated with 100 µg/ml NB54 for 5 days, we observed insignificant differences in *MECP2*_e1 and _e2 expression before and after NB54 treatment (less than 25%, Student's t test p>0.05) and could be attributed to XCI variability (Fig. 3), thereby supporting the notion that NB54 has no effect on the mRNA level.

### Functionality of MeCP2 after NB54 treatment

**Figure 4:**
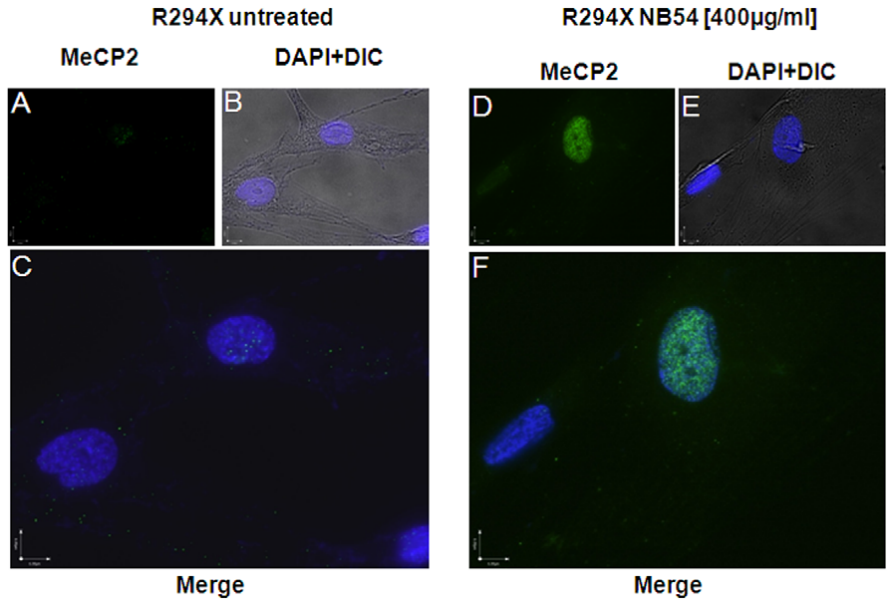
NB54 treatment in RTT fibroblasts recovers full-length MeCP2 at the nucleus. Immunofluorescence studies of untreated (A–C) and NB54-treated (D–F) R294X fibroblasts, using MeCP2 C-terminal antibody. Staining of the full-length MeCP2 (green signal in D, F) corresponds with the DAPI staining (blue signal in E, F), thus indicating its nuclear localization.

**Figure 5:**
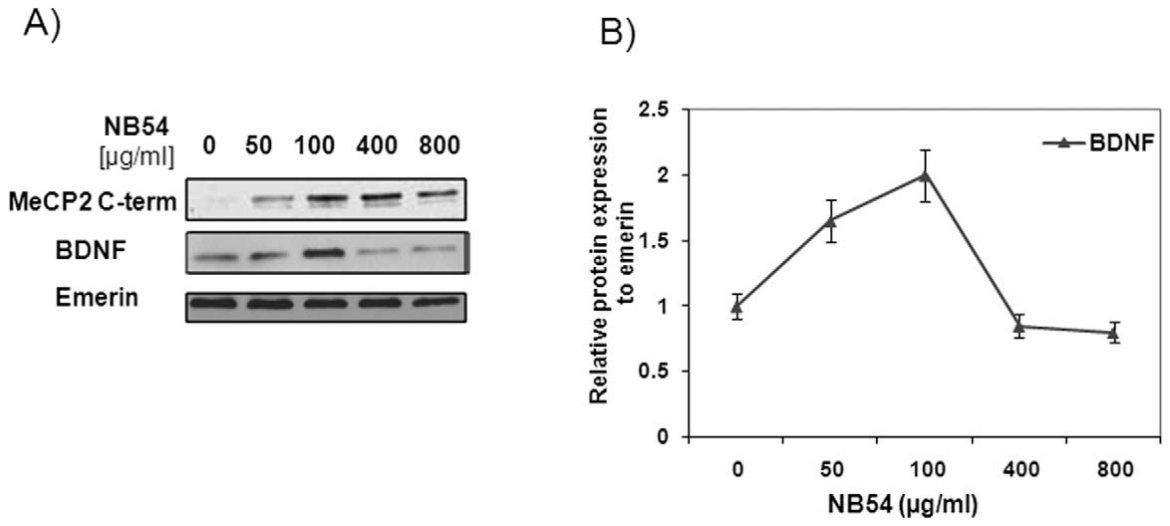
NB54 treatment increases BDNF levels in R294X fibroblasts. (A) Western blots of MeCP2, BDNF and emerin proteins after NB54 treatment (50–800 µg/ml for 5days) in R294X fibroblasts; (B) Graph indicates mean ± SEM of BDNF expression levels normalized to emerin at different NB54 concentrations derived from three independent experiments (n = 3).

Studying localization of the recovered full-length MeCP2 in NB54-treated R294X fibroblasts by immunofluorescence with MeCP2 C-terminal antibody (green), we found that MeCP2 was targeted to the cell nucleus (DAPI blue) (Fig. 4), as expected. In R294X fibroblasts treated with elevating concentrations of NB54 between 50 and 800 µg/ml, we also looked at the levels of MeCP2 and BDNF (Fig. 5A). We found dose-dependent recovery of the full-length MeCP2 with increasing concentrations of NB54, reproducing our previous findings with the same mutation (Fig. 1A), a negligible satellite band could be due to non-specificity of the MeCP2 antibody. More importantly, we found parallel increase in BDNF levels with 2-fold maximum at 100 µg/ml NB54, which decreased at higher drug concentrations (Fig. 5B).

## Discussion

Recent insights from RTT animal model suggest that neuronal dysfunction resulting from MeCP2 deficiency can be reversed, even in the adult mice, once MeCP2 is recovered [21], [22]. These extraordinary findings prompt further efforts in developing pharmacological approaches targeted at enhancement of MeCP2 levels and function in the CNS. As *MECP2* nonsense mutations that lead to premature translational termination and truncated MeCP2 are relatively common in RTT, pharmacological suppression of these mutations by small molecules, such as aminoglycosides, could be potentially promising for human patients. So far, the most substantial experience has been obtained with gentamicin in restoring the expression of functional dystrophin and cystic fibrosis transmembrane conductance regulator (CFTR) proteins in mouse models [28], [29] and in human patients [36], [37]. Especially encouraging recent reports indicated that enhancement from 1% to only 5% of normal CFTR levels may greatly reduce the severity or eliminate principal manifestations of cystic fibrosis [30], [38]. However, severe ototoxicity and nephrotoxicity of gentamicin [32], [33] and its reduced read-through efficiency at subtoxic doses, as well as its limited permeability through the blood-brain-barrier [32], have limited its clinical use [30], specifically for CNS disorders. Currently, only a limited number of aminoglycosides, including gentamicin, amikacin, and tobramycin, are in clinical use as antibiotics for internal administration in humans, among these, tobramycin does not have nonsense suppression activity.

The newly developed aminoglycoside NB54 has been demonstrated as both, appreciably more effective in nonsense suppression and less toxic than gentamicin [35], enabling read-through of specific nonsense mutations underlying important human genetic disorders, i.e. Usher syndrome, cystic fibrosis, Duchenne muscular dystrophy and Hurler syndrome and being 8-fold less toxic than gentamicin (LD50 = 500 mg/kg *vs.* 60 mg/kg). Because of this precedent and recent studies of gentamicin suppressing *MECP2* nonsense mutations *in vitro* using transfected HeLa [31] and HEK293 [39] cell lines, we studied NB54 and gentamicin effects *ex vivo* in primary fibroblasts derived from classical RTT patients. Apart from R255X, we included the three most common RTT causative *MECP2* nonsense mutations, R294X, R270X and R168X, all resulting in UGA stop codon that was demonstrated as the most effective gentamicin read-through target [40]. Most recent study also tested NB aminoglycosides in mouse fibroblasts harboring *MECP2* R168X mutation [41].

For all the three RTT nonsense mutations studied here, NB54 had better suppression effect than gentamicin in enabling to achieve higher expression levels and higher read-though efficiency of the full-length MeCP2 at lesser concentrations. NB54 effect was mutation specific with peak efficiencies at 100 µg/ml in R270X and R168X, and simple dose-dependent relationship in R294X fibroblasts. The most significant NB54 effect was observed in R168X (38% read-through efficiency) compared to R270X (27%) and R294X (18%). The best read-through for gentamicin was obtained in R294X (24%) compared to R270X (15%) and R168X (18%) fibroblasts, thus reproducing previous studies of gentamicin effect for the same *MECP2* mutations in transfected HeLa and HEK293 lines [31], [39]. Apart from R294X fibroblasts, both drugs had lesser efficiencies at concentrations higher that 100 µg/ml, suggesting that both are toxic, but gentamicin was more toxic than NB54 at the same concentrations, also evident from our cell toxicity data (Table 1). Serum gentamicin concentrations above 12 µg/ml have been previously shown as toxic for clinical use. In order to facilitate higher read-through effect, we experimented with MeCP2 recovery using drug concentrations up to 800 mg/ml and longer exposure time (5 days). It is of note that the emerin expression levels do not reflect cytotoxicity to the same extent as the cell counts, NB54 effect on emerin expression was minimal (Figure 1A–C), emerin being frequently used in Western blot analyses as internal control for nuclear protein variation [42].

The issue of the significance of nucleotide context, particularly the position +4 immediately following the stop codon, in dictating the read-through efficiency has not been entirely resolved. Several studies suggested that the gentamicin permissive read-through is affected by the hierarchy C>U>G≥A at this position [36], [40], while other studies did not support their findings [43], [44]. The present study supports the notion that the nature of nucleotide +4 may have contributing effect on NB54-induced read-through of *MECP2* nonsense mutations, as R168X (UGA G) had higher read-through than R270X (UGA A) and than R294X (UGA U) (Fig. 2). Effects of other factors, especially those involved in mRNA stabilization and escape from nonsense mediated mRNA decay (NMD) [44], [45] cannot be ruled out and should be investigated in future studies. Additionally, as aminoglycosides mechanism of action involves binding to the 16S rRNA and interfering with the ribosomal proofreading [27], [46], we suggest that NB54 does not affect the expression of normal MeCP2 allele (25–50% background levels) and that the observed increase in MeCP2 levels is predominantly resulting from suppression of the MeCP2 nonsense allele. Due to the same reason, NB54 treatment did not significantly affect the expression levels of both *MECP2*_e1 and _e2 transcripts. We have previously reported that *MECP2* truncating mutations (nonsense or frame-shift deletions) are associated with lower *MECP2* RNA levels in peripheral blood possibly due to the nonsense mediated mRNA decay mechanism [47], suggesting that the truncated MeCP2 protein should be also reduced in RTT fibroblasts.

One of the limitations of this study is variability of the background MeCP2 expression (no drug), which makes the interpretation and comparisons between various drug effects difficult (Fig. 1A–C). This phenomenon has been known from other studies of premature termination codons (PTC) in CFTR and dystrophin genes [44], [48]. In our experiments, this problem is accentuated by XCI variability in primary fibroblasts from RTT heterozygous females, thereby necessitating standardization of the read-through efficiency considering background MeCP2 expression (no drug), fully active MeCP2 reference (HFF) and emerin. Future studies may resolve this drawback using single-cell subcloning of primary RTT fibroblasts, thus enabling to compare between expression levels of the active and non-active MeCP2 alleles, as well as the normal and truncated MeCP2. This approach has already been used for other *MECP2* mutations in female RTT fibroblasts [49]. Hemizygous RTT male fibroblasts harboring *MECP2* nonsense mutation could also be helpful in resolving this question, but such patients are extremely rare and are often misdiagnosed.

Our maybe most interesting finding has been to show that NB54 treatment at 100 µg/ml induces up to 2-fold increase in the BDNF levels, which was reduced at higher NB54 concentrations (400–800 µg/ml) probably due to toxic effects. BDNF is the one of the most important MeCP2 targets, being crucially involved both, in synaptic plasticity during brain development [50] and in adults [51]. Mechanism of MeCP2 action on BDNF expression is not entirely clear, since it was shown that active MeCP2 represses BDNF *in vitro* [14], [15], however brain Bdnf was decreased *in vivo* in Mecp2-null mice [52], [53], and was up-regulated in MeCP2-overexpressing mice [16]. In addition, BDNF and other MeCP2-dependent factors were up-regulated after partial rescue of MeCP2 deficiency *in vitro* using HDAC inhibitors [42]. Regardless of their relationship, BDNF enhancement in Mecp2-deficient mice was shown to significantly improve the RTT-like phenotype and even to enable its partial rescue [52]. Although the role BDNF in fibroblasts is not clear, our findings of concomitant increase in the full-length MeCP2 and BDNF levels resulting from NB54 treatment of RTT fibroblasts suggest that the recovered MeCP2 may have retained its functional properties, which is moreover supported by its appropriate nuclear translocation.

In summary, this study proposes NB54 as a potential new therapeutic agent for RTT nonsense mutations, and in so doing supports the “proof of principle” that some RTT causative *MECP2* nonsense mutations can be at least partially suppressed by less toxic aminoglycosides and aminoglycoside mimetics. This therapeutic approach is particularly attractive for RTT, as aminoglycosides are probably acting only on the mutated allele and thus enabling to avoid MeCP2 over-expression. In addition, aminoglycosides mechanism of action is not dependent on knowing the exact MeCP2 function and underlying biology. Another potentially interesting agent with a capability to suppress premature protein termination without obvious side effects, is PTC124 [54], although its effects on CNS disorders including RTT are yet to be explored. In the same way, further studies of NB54 effects should be carried out in mouse model with one of the *MECP2* nonsense mutations looking at NB54 permeability through the blood-brain barrier, distribution in brain tissue and recovery of RTT symptoms.

## Methods

### Patient selection

RTT patients were recruited from the Israeli Rett Center operating at the Safra Pediatric Hospital at the Sheba Medical Center, providing clinical and genetic diagnoses for RTT as well as ongoing clinical follow up. Study rationale was explained to the patients' legal guardians, after which they signed informed consent for skin biopsy extraction approved by the Helsinki Committee at Sheba Medical Center and by the Israeli Ministry of Health.

### Primary fibroblasts culture and treatment

Fibroblasts were expanded from patients' skin biopsies and cultured in Dulbecco's modified Eagle's medium (Biological Industries, Israel) supplemented with 10% fetal calf serum (FCS), 2 mM L-glutamine, 100 mg/ml streptomycin and 100 units/ml penicillin (Biological Industries) at 37°C in humidified incubator with 5% CO_2_. Human foreskin fibroblasts (HFF) used as reference for normal MeCP2 expression were cultured in the same conditions. Gentamicin sulfate (Biological Industries) or NB54 sulfate were added into the medium in concentrations ranging from 50 to 800 µg/ml and cells were grown for 3 to 5 days.

### RNA extraction and real-time quantitative PCR

Total RNA was extracted using Trizol reagent (Invitrogen, USA) according to manufacturer's protocol. First-strand cDNA was generated from 1 µg total RNA in the presence of random hexamer primers using High Capacity cDNA reverse transcription kit (Applied Biosystems, USA). Gene expression was quantified by real-time PCR using SYBR green PCR mix (Kapa Biosystems, USA) in the presence of specific primers for *MECP2*_e1 and _e2 isoforms [10] compared to the *GAPDH* gene. Signals were analyzed on ABI Prism 7900 SDS (Applied Biosystems). All the reactions were performed in triplicates and means were compared using Student's t-tests.

### Nuclear protein extraction

Cells were harvested in ice-cold PBS, washed twice and centrifuged. Nuclear proteins were extracted from cell pellets using NucBuster protein extraction kit (Merk, Germany) and re-suspended according to manufacturer's protocol. Protein concentration was determined by Bradford modified method (BCA Protein Assay; Pierce, USA) and equal amounts of proteins were subjected to Western Blot analysis.

### Western blot analysis

Proteins were separated on 12% SDS-PAGE, transferred to nitrocellulose membrane (iBlot, Invitrogen) and detected using ECL SuperSignal West Pico Chemiluminescent Substrate (Thermo Scientific, USA) and CL-XPosure X-ray films (Thermo Scientific). Primary antibodies included: rabbit mouse monoclonal C-terminal anti-MeCP2 (Mec-168, Abcam, UK), rabbit polyclonal anti-BDNF (Alomone Labs Ltd, Israel), rabbit polyclonal anti-emerin (Santa Cruz Biotec, USA) and goat polyclonal anti-actin (Santa Cruz Biotec). Secondary antibodies were: peroxidase-conjugated goat anti-rabbit, goat anti-mouse and donkey anti-goat (Jackson ImmunoResearch Laboratories, USA) diluted 1∶10,000. Read-through efficiency was calculated by comparing expression of the full-length C-terminal containing MeCP2 in RTT and HFF fibroblasts, using digital densitometry by EzQuant software (EzQuant Ltd, Israel). MeCP2 read-through efficiency data was obtained from three independent experiments for each mutation and at least two Western blots for each experiment; means±SEM were compared using Student's t-tests and statistically significant differences (p<0.05) were indicated (*).

### Immunofluorescence analysis

Cells were grown on coverslips and fixed with 4% paraformaldehyde in PBS for 20 min. Permeabilization included incubation with 0.1% Triton X-100 for 5 min, washing with TBS (100 mM Tris-HCl pH 7.5, 150 mM NaCl) and blocking with 5% skimmed milk in TBS containing 0.1% Tween 20 (TBS-T) for 30 min, all at room temperature. Incubations with primary antibody (C-terminal anti-MeCP2, Abcam; 1∶1000 dilution) and secondary antibody (conjugated goat anti-mouse Alexa Fluor 488, Invitrogen; 1∶500 dilution) were performed in blocking solution for 1 h each, washing with TBS-T between and after incubations. Cells were incubated before mounting with 4′,6-diamineo-2-phenylindole (DAPI) with antifade 1∶10,000 dilution (Sigma-Aldrich, USA). Cells were photographed using Improvision optic grid (Improvision, UK) acquisition and fluorescent microscope (Olympus, USA).
